# Supplementary material for: Porin A and α/β-hydrolase are necessary and sufficient for hemolysis induced by Bartonella bacilliformis
Source: Nat Commun. 2025 Nov 28;16:10809. doi: 10.1038/s41467-025-66781-x (PMC12669792; doi:10.1038/s41467-025-66781-x)

Supplementary Tables

Supplementary table 1: Percent identity matrix of porin A homologues in other *Bartonella* species created by Clustal2.1.

**porin A**	*B. bacilliformis* (WP_005766494.1)	*B. henselae* (WP_011181078.1)	*B. quintana* (WP_011179682.1)	*B. tribocorum* (WP_100130715.1)
B. bacilliformis (WP_005766494.1)	100.00			
B. henselae (WP_011181078.1)	64.57	100.00		
B. quintana (WP_011179682.1)	64.48	82.29	100.00	
B. tribocorum (WP_100130715.1)	60.30	78.25	77.94	100.00

Supplementary table 2: Percent identity matrix of α/β- hydrolase homologues in other *Bartonella* species created by Clustal2.1.

**α/β-hydrolase**	*B. bacilliformis* (WP_005766862)	*B. henselae* (WP_034447759.1)	*B. quintana* (WP_034449243.1)	*B. tribocorum* (WP_100129362.1)
B. bacilliformis (WP_005766862)	100.00			
B. henselae (WP_034447759.1)	79.70	100.00		
B. quintana (WP_034449243.1)	79.70	91.37	100.00	
B. tribocorum (WP_100129362.1)	79.75	89.34	89.85	100.00

Supplementary table 3: Pairwise deduced protein sequence alignment of porin A using 15 closed *B. bacilliformis* genomes. The values are given in percent.

B. bacilliformis	1	2	3	4	5	6	7	8	9	10	11	12	13	14	15
KC583 1															
KC584 2	100														
INS 3	100	100													
San Pedro600-02 4	100	100	100												
USM-LMMB06 5	100	100	100	100											
Peru-18 6	99,26	99,26	99,26	99,26	99,26										
CUSCO5 7	99,26	99,26	99,26	99,26	99,26	100									
Peru38 8	99,26	99,26	99,26	99,26	99,26	100	100								
Ver075 9	99,26	99,26	99,26	99,26	99,26	100	100	100							
USM-LMMB07 10	99,26	99,26	99,26	99,26	99,26	100	100	100	100						
Heidi Mejia 11	100	100	100	100	100	99,26	99,26	99,26	99,26	99,26					
Hosp800-02 12	100	100	100	100	100	99,26	99,26	99,26	99,26	99,26	100				
VAB9028 13	100	100	100	100	100	99,26	99,26	99,26	99,26	99,26	100	100			
CAR600-02 14	100	100	100	100	100	99,26	99,26	99,26	99,26	99,26	100	100	100		
Cond044 15	96,55	96,55	96,55	96,55	96,55	96,55	96,55	96,55	96,55	96,55	96,55	96,55	96,55	96,55	

Supplementary table 4: Pairwise deduced protein sequence alignment of α/β-hydrolase using 15 closed *B. bacilliformis* genomes. The values are given in percent.

B. bacilliformis	1	2	3	4	5	6	7	8	9	10	11	12	13	14	15
KC583 1															
KC584 2	100														
INS 3	100	100													
San Pedro600-02 4	100	100	100												
USM-LMMB06 5	100	100	100	100											
Peru-18 6	100	100	100	100	100										
CUSCO5 7	100	100	100	100	100	100									
Peru38 8	100	100	100	100	100	100	100								
Ver075 9	100	100	100	100	100	100	100	100							
USM-LMMB07 10	100	100	100	100	100	100	100	100	100						
Heidi Mejia 11	99,75	99,75	99,75	99,75	99,75	99,75	99,75	99,75	99,75	99,75					
Hosp800-02 12	99,75	99,75	99,75	99,75	99,75	99,75	99,75	99,75	99,75	99,75	100				
VAB9028 13	99,75	99,75	99,75	99,75	99,75	99,75	99,75	99,75	99,75	99,75	100	100			
CAR600-02 14	99,75	99,75	99,75	99,75	99,75	99,75	99,75	99,75	99,75	99,75	100	100	100		
Cond044 15	99,75	99,75	99,75	99,75	99,75	99,75	99,75	99,75	99,75	99,75	99,5	99,5	99,5	99,5	

Supplementary table 5: Predicted physical and chemical parameters analyzed by the ExPASy ProtParam tool.

	molecular weight (Da)	theoretical PI	extinction coefficient (M-1 cm-1)	instability index	aliphatic index	grand average of hydro-pathicity (GRAVY)
porin A	44,609	8.16	86,875	19.97, stable	79.33	-0.272
α/β-hydrolase	44,866	9.37	37,945	30.50, stable	88.81	-0.117

Supplementary table 6: Subcellular localization and signal sequences predicted by PSORT, cello, LocTree3 and SignalP.

	sub-cellular localization			signal peptide
	PSORTb	cello	LocTree3	
porin A	outer membrane	outer membrane	outer membrane	signal peptide (Sec/SPI) pos. 1-22
α/β-hydrolase	unknown	periplasm	secreted	lipoprotein signal peptide (Sec/SPII) pos. 1-29

Supplementary table 7: Protein classification and identification of protein function by motif and domain analysis predicted by Pfam, Interproscan and Conserved Domain Search (CDD).

	CDD	Interproscan	Pfam
porin A	OM_channels superfamily (10495026)	porin, alpha proteobacteria type (IPR003684)	outer membrane beta-barrel protein superfamily (CL0193), porin_2 (PF02530)
α/β-hydrolase	esterase/lipase superfamily (COG4782)	α/β-hydrolase fold (IPR029058), protein of unknown function DUF900 (IPR010297)	α/β-hydrolase of unknown function (CL0028), DUF900 (PF05990)

Supplementary table 8: Phospholipase inhibitor library (Merck, Darmstadt, Germany).

no.	name	CAS number	Hill formula
1	neomycin sulfate	1405-10-3	$C_{23}H_{46}N_6O_{13} \cdot xH_2SO_4$
2	cytidine 5'-diphosphocholine sodium	33818-15-4	$C_{14}H_{25}N_4NaO_{11}P_2 \cdot 2H_2O$
3	compound 48/80	94724-12-6	$C_{32}H_{45}N_3O_3$
4	N-(p-Amylcinnamoyl) anthranilic acid	110683-10-8	$C_{21}H_{23}NO_3$
5	autotaxin inhibitor II, HA130	1229652-21-4	$C_{24}H_{19}BFNO_5S$
6	ML298 [3,4-Difluoro-N-[2-[1-(3-fluorophenyl)-4-oxo-1,3,8-triazaspiro[4.5]dec-8-yl]ethyl]-benzamide]	1426916-02-0	$C_{22}H_{23}F_3N_4O_2$
7	varespladib	172732-68-2	$C_{21}H_{20}N_2O_5$
8	aristolochic acid I	313-67-7	$C_{17}H_{11}NO_7$
9	cPLA2a Inhibitor II, pyrrophenone	341973-06-6	$C_{49}H_{37}F_2N_3O_5S_2$
10	ABO dihydrochloride 6-amino-2, 3-dihydro-3-hydroxymethyl-1, 4-benzoxazine	896126-03-7	$C_9H_{12}N_2O_2 \cdot 2HCl$
11	U73122 hydrate	112648-68-7	$C_{29}H_{40}N_2O_3 \cdot xH_2O$
12	VO-OHpic trihydrate	476310-60-8	$C_{12}H_9N_2O_8V \cdot H \cdot 3H_2O$
13	cinnamycin	110655-58-8	$C_{89}H_{125}N_{25}O_{25}S_3$
14	FIPI hydrochloride hydrate	939055-18-2	$C_{23}H_{24}FN_5O_2 \cdot HCl \cdot xH_2O$
15	MJ 33	199106-13-3	$C_{22}H_{43}F_3O_6PLi$
16	ONO-RS-082	99754-06-0	$C_{21}H_{22}ClNO_3$
17	D609, potassium salt	83373-60-8	$C_{11}H_{15}OS_2 \cdot K$
18	aristolochic acid sodium salt	10190-99-5	$C_{17}H_{10}NNaO_7$
19	bromoenol lactone	88070-98-8	$C_{16}H_{13}BrO_2$
20	spermine, tetrahydrochloride	306-67-2	$C_{10}H_{26}N_4 \cdot 4HCl$
21	sPLA2-IIA Inhibitor I	236394-37-9	$C_{41}H_{50}N_8O_6 \cdot xC_2HF_3O_2$
22	ASB14780	1069046-00-9	$C_{31}H_{27}NO_3 \cdot C_4H_{11}NO_3$
23	U73343	142878-12-4	$C_{29}H_{42}N_2O_3$
24	ET-18-OCH3	70641-51-9	$C_{27}H_{58}NO_6P$
25	HQL 79	162641-16-9	$C_{22}H_{27}N_5O$
26	PLD inhibitor, FIPI	1781834-93-2	$C_{23}H_{24}FN_5O_2$
27	autotaxin inhibitor I, S32826	1103672-43-0	$C_{21}H_{34}NNa_2O_4P \cdot 2H_2O$

Supplementary table 9: Bacterial strains and plasmids used in this study.

	**characteristics**	**reference**
**bacteria**		
B. bacilliformis KC583	wildtype	ATCC® 35685^{TM}, Manassas, VA, USA
B. bacilliformis KC584	wildtype	ATCC® 35686^{TM}, Manassas, VA, USA
E. coli NEB 5 alpha	*fhuA2 a(argF-lacZ)U169 phoA glnV44 a80a(lacZ)M15 gyrA96 recA1 relA1 endA1 thi-1 hsdR17*	NEB, Ipswich, MA, USA
**plasmids and transposon**		
pBIISK_*sacB/kanR*	*sacB,* Kan^R	according to Stahl *et al.*, 2015 [1]
pBBR1MCS-2	bacterial shuttle vector*,* Kan^R	Addgene, Watertown, MA, USA
EZ-Tn5	Kan^R	Epicentre, Madison, WI, USA

Supplementary table 10: Primers used in this study. Bold letters indicate overhangs and underlined letters BamHI cutting sites. Primers were constructed in Geneious Prime (Dotmatics, Boston, MA, USA) and synthesized at Metabion (Planegg, Germany).

**Identification of transposon insertion sites**		
**primer designation**	**sequence (5'→3')**	**function**
Tn5_F	CAACAAAGCTCTCATCAACC	sequencing of Tn5 insertion site
Tn5_R	TTGAATATGGCTCATAACACC	
Kan2-FP2	GATCCTCTAGAGTCGACCTG	
Kan2-RP1	GCAATGTAACATCAGAGATTTTGAG	
**Generation of deletion plasmids, verification of genomic deletions**		
**primer designation**	**sequence (5'→3')**	**function**
pBIISK_sacB_F	CTAGTTCTAGAGCGGCCGCCACC	linearisation of pBIISK_sacB
pBIISK_sacB_R	TGGATCCCCCGGGCTGCAGG	
pBIISK_seq_F	ATTGGTTGTAACACTGGCAGAG	sequencing of pBIISK_sacB inserts
pBIISK_seq_R	CTTTAGTGAGGGTTAATTGCGC	
porA_down_F	**CTGAAGGCGTATC**GCAAGGTTTTAATATTCAT	amplification of *porA* downstream fragment with upstream-homologous overhangs
porA_down_R	**GCGGTGGCGGCCGCTCTAGAACTAG**GGTGCAAAAAGCTTCTTCCC	
porA_up_F	**GAATTCCTGCAGCCCGGGGGATCCA**ACTGGTGTCAGGTCTTTACC	amplification of *porA* upstream fragment with downstream-homologous overhangs
porA_up_R	**TTAAAACCTTGC**GATACGCCTTCAGCGTTC	
porA_del_seq_R	GACGCATAAACGGTCTTAGC	sequencing of the inserted *porA* fragment in pBIISK_sacB and of the genomic deletion of *porA*
α/β-hyd-down_F	**GATGGTGTTACGGTGAAA**TTGTTCTCTGTGGTTGATTT	amplification of *hyd* downstream fragment with upstream-homologous overhangs
α/β-hyd-down_R	**GCGGTGGCGGCCGCTCTAGAACTAG**CCCAACGACAAATATAGACGC	
α/β-hyd-up_F	**GAATTCCTGCAGCCCGGGGGATCCA**GCAGCATGTTTGAGGATTG	amplification of *hyd* upstream fragment with downstream-homologous overhangs
α/β-hyd-up_R	**ATCAACCACAGAGAACA**ATTTCACCGTAACACCATC	
α/β-hyd_del_seq_F	GTGGAAGTTCGAGTCTTCTC	sequencing of the inserted *hyd* fragment in pBIISK_sacB and of the genomic deletion of the α/β-hydrolase

Generation and verification of complementation plasmids

primer designation	sequence (5'→3')	function
M13_F	**GTAAAACGACGGCCAGT**	sequencing of MCS of pBBR1MCS2
M13_R	**CAGGAAACAGCTATGAC**	
porA_comp_F	**TAAGCAGGATCC**GACGCATAAACGGTCTTAG	amplification of *porA* with BamHI site for pBBR1MCS-2 cloning
porA_comp_R	**TGCTTAGGATCCC**GTATAGCTAGCAAGACATG	
α/β-hyd_comp_F	**TAAGCAGGATCC**ACTCTCCATTATCGCTTAAC	amplification of *hyd* with BamHI site for pBBR1MCS-2 cloning
α/β-hyd_comp_R	**TGCTTAGGATCC**CACAAACTCACTTAATGACTG	
porA_α/β-hyd_comp_F1	**TCATTAAGTGAGTTTGTG**CTGCAGGAATTCGATATCAAGC	linearisation of pBBR1MCS-2
porA_α/β-hyd_comp_R1	**TAAGACCGTTTATGCGTC**GGATCCACTAGTTCTAGAGC	
porA_α/β-hyd_comp_F2	**TCTAGAACTAGTGGATCC**GACGCATAAACGGTCTTAGC	*porA* amplification with overhang to pBBR1MCS-2 and to the *hyd* insert
porA_α/β-hyd_comp_R2	**AAGCGATAATGGAGAGTG**CGTATAGCTAGCAAGACATG	
porA_α/β-hyd_comp_F3	**TGTCTTGCTAGCTATACG**CACTCTCCATTATCGCTTAAC	*hyd* amplification with overhang to the *porA* insert and to pBBR1MCS-2
porA_α/β-hyd_comp_R3	**GATATCGAATTCCTGCAG**CACAAACTCACTTAATGACTGG	
porA_ α/β-hyd_seq_F	CTTCTGTAAAGCGGCAGC	sequencing primer for *porA* and *hyd* complementation plasmid
porA_ α/β-hyd_seq_R	GCTATTACGCCAGCTGGC	

RT-PCRs

primer designation	sequence (5'→3')	function
rpod_1F	GGTGTAGGTTCGCGCCCAAT	*rpoD* RT-PCR
rpod_1R	CAAGGCCGTGAATTGGATTCAG	
porA_RT-PCR_ F	CACGCGGCCCCAGATATTGT	*porA* RT-PCR
porA_RT-PCR_ R	GGCATTGCCCAATTCAGCTCCG	
α/β-hyd_RT-PCR_F	GGTACATTTCGTGCAGCTC	*hyd* RT-PCR
α/β-hyd_RT-PCR_R	GGCAAATACATTATGGGCTCC	

Site-directed mutagenesis		
**primer designation**	**sequence (5'→3')**	**function**
S205A_F	TATCGCACATGCGATGGGCAATTTTG	site-directed mutagenesis of Ser205 to Ala in the catalytic triad of the α/β-hydrolase
S205A_R	ACAGAAATTTGATCAGCATTCG	
D267N_F	ATCTCGTAAGAACAAAGCTCTTGC	site-directed mutagenesis of Asp267 to Asn in the catalytic triad of the α/β-hydrolase
D267N_R	ACCAAAACAGCTGTTG	
H310A_F	TGGTGGAGCCGCGAATGTATTTGCC	site-directed mutagenesis of His310 to Ala in the catalytic triad of the α/β-hydrolase
H310A_R	TCAACGTTAGAGACATCAAG	
S205A_D267N_F	TATCGCACATGCGATGGGCAATTTTG	site-directed mutagenesis of Ser205 to Ala, Asp267 to Asn and His310 to Ala in the catalytic triad of the α/β-hydrolase
S205A_D267N_R	GCAAGAGCTTTGTTCTTACGAGAT	
D267N_H310A_F	ATCTCGTAAGAACAAAGCTCTTGC	site-directed mutagenesis of Ser205 to Ala, Asp267 to Asn and His310 to Ala in the catalytic triad of the α/β-hydrolase
D267N_H310A_R	GGCAAATACATTCGCGGCTCCACCA	
H310A_S205A_F	TGGTGGAGCCGCGAATGTATTTGCC	site-directed mutagenesis of Ser205 to Ala, Asp267 to Asn and His310 to Ala in the catalytic triad of the α/β-hydrolase
H310A_S205A_R	CAAAATTGCCCATCGCATGTGCGATA	
**Generation of truncated α/β-hydrolase**		
**primer designation**	**sequence (5'→3')**	**function**
hyd_ΔCT_F	**ATCTGCTTCCTATATACTTTTA**TTGCATAATTGTCGAGGATA	amplification of a C-terminally truncated *hyd* version with overhang to pBBR1MCS-2
hyd_ΔCT_R	**TATCCTCGACAATTATGCAA**TAAAAGTATATAGGAAGCAGAT	

Supplementary Figures

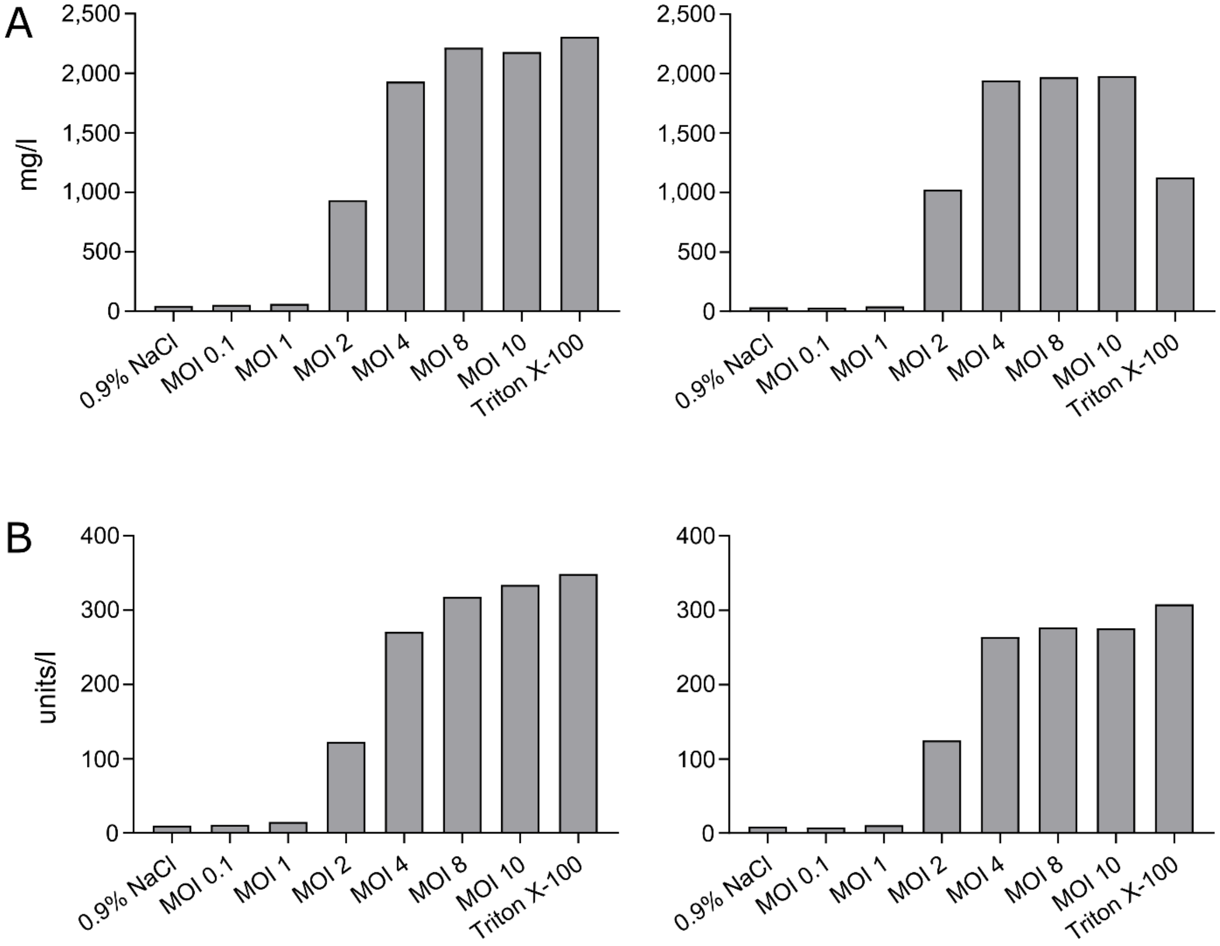

Supplementary figure 1. Release of hemoglobin and lactate dehydrogenase from *B. bacilliformis*-infected erythrocytes. Freshly isolated human erythrocytes (from two donors) were washed three times and infected with *B. bacilliformis* strain KC583 (MOIs: 0.1, 1, 2, 4, 8, 10) for 20 hours. Negative control: erythrocytes incubated in 0.9% NaCl without bacteria, positive control: erythrocytes treated with 1% (v/v) Triton X-100. Two independent experiments have been replicated (both depicted here). **(A)** Free hemoglobin (mg/l) and **(B)** released lactate dehydrogenase (units/l) quantified from the supernatants.

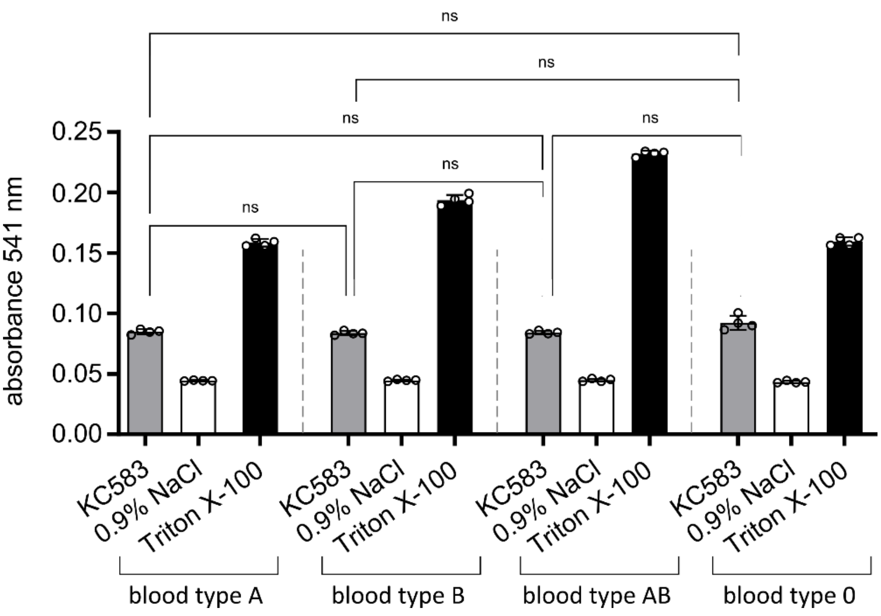

Supplementary figure 2. Susceptibility of erythrocytes from different blood groups to *B. bacilliformis*-induced hemolysis. Erythrocytes from blood groups A, B, AB, and 0 (grey bars) were infected with *B. bacilliformis* (MOI: 5) and hemolysis was quantified photometrically at 541 nm after 20 hours. Negative controls (white bars): erythrocytes incubated in 0.9% NaCl without bacteria, positive controls: erythrocytes treated with 1% (v/v) Triton X-100 (black bars). All samples were run in quadruplicate (n = 4). Data are presented as mean values ± SD. Statistical significance between the blood groups (A, B, AB, 0) was determined by one-way ANOVA (F (3, 12) = 5.853, $p < 0.011$, $R^2 = 0.594$), followed by Tukey's multiple comparison test ($p \geq 0.01$: not significant, ns). Two independent experiments have been replicated.

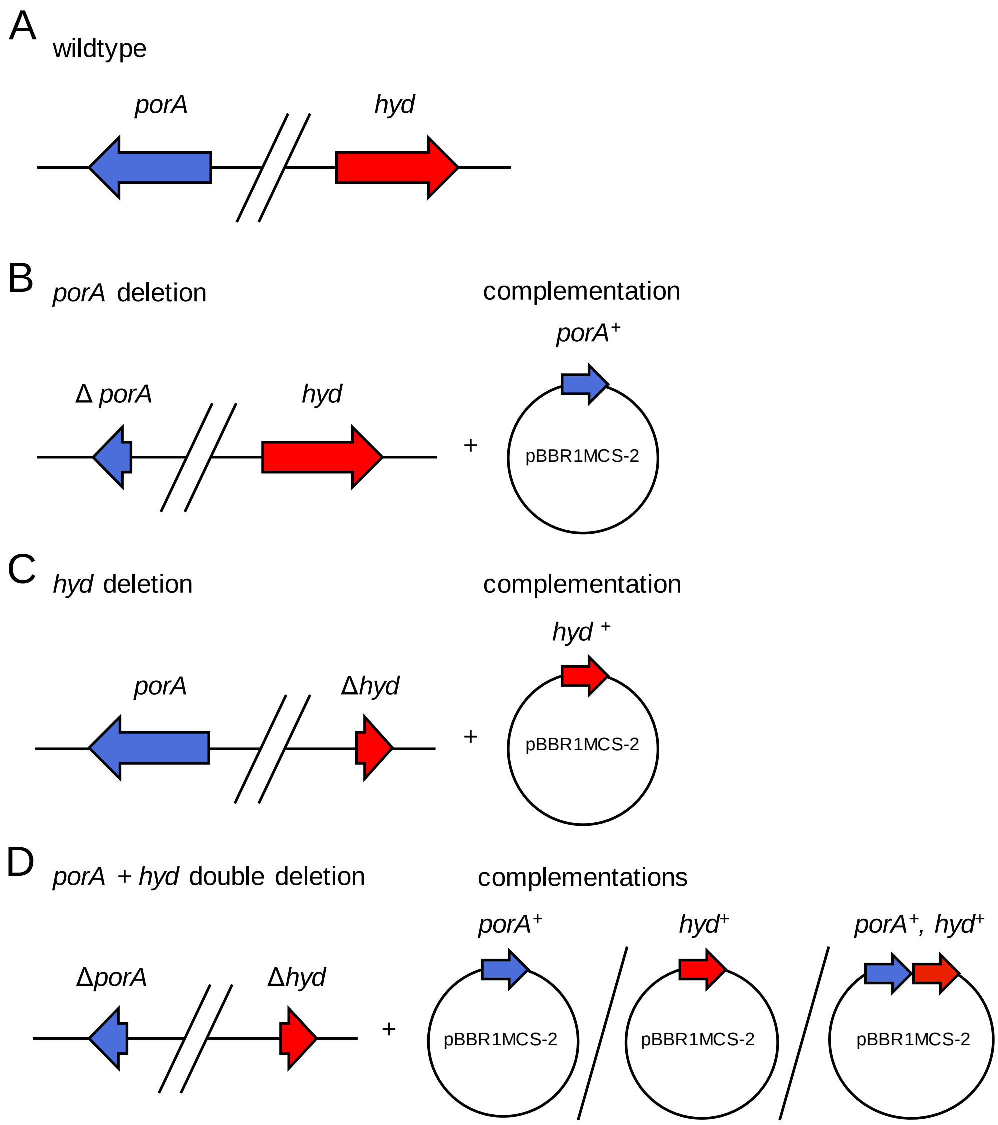

Supplementary figure 3: Generation of *B. bacilliformis* mutants. Markerless targeted mutagenesis was performed to generate Δ*porA* and Δ*hyd* deletion mutants (**A:** wildtype, **B, C:** single deletions, **D:** double deletions). For complementation, the respective genes along with their native promoters were subsequently introduced via a vector plasmid (**B**, **C**, **D**: single and double complementation).

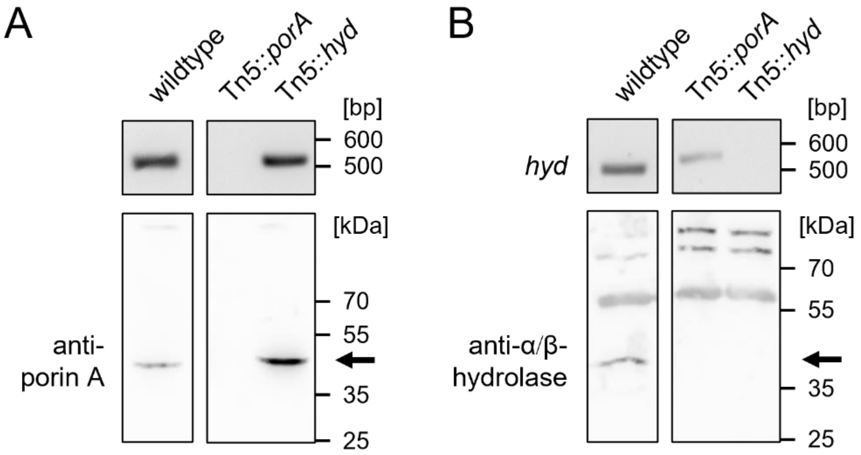

Supplementary figure 4: Western blot analysis of hemolysis deficient transposon mutants. Analysis of porin A and α/β-hydrolase expression by RT-PCR (top panel) and Western blot analysis (bottom panel) using porin A or α/β-hydrolase-specific primers and antibodies. Notably, α/β-hydrolase is not detected by Western blotting in the Tn5::*porA* mutant despite expression of *hyd* mRNA (as demonstrated by RT-PCR). Two independent experiments have been replicated.

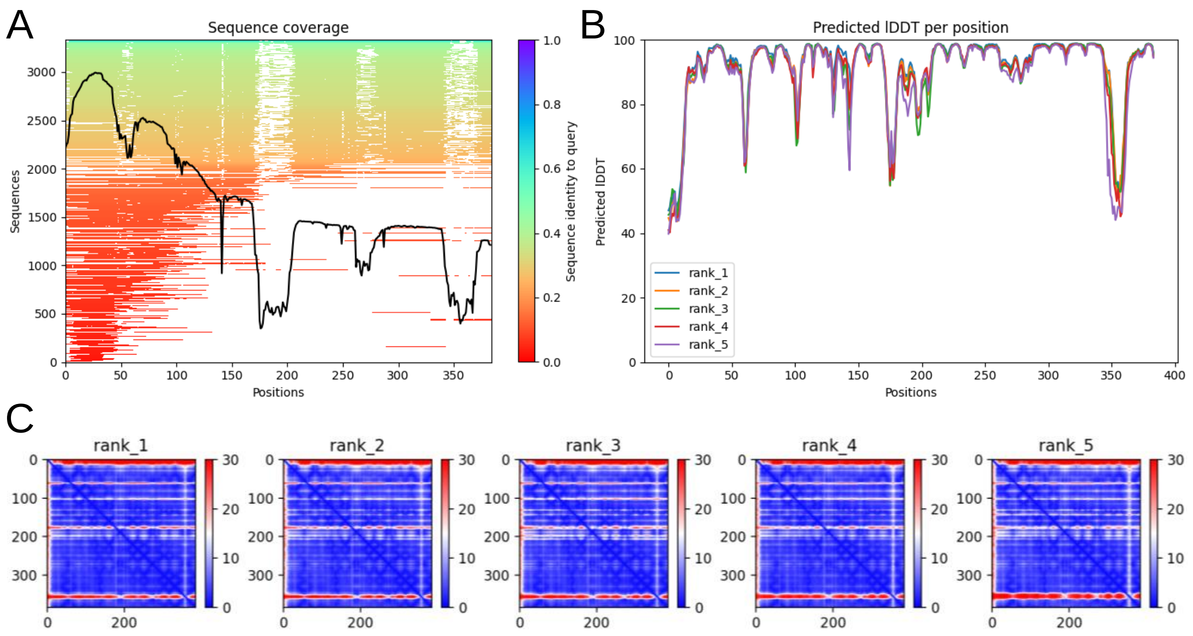

Supplementary figure 5: Quality analysis of the porin A structure prediction. (**A**) Multiple sequence alignment for porin A. Shown are the sequence coverage and sequence identity of homologous structural templates per amino acid used for Alphafold2 structure prediction. (**B**) pLDDT scores of porin A. Plotted are the pLDDT scores per amino acid position for the five calculated porin A models. The model with the highest pLDDT score was selected for further analysis (blue, rank_1). (**C**) Predicted aligned error (PAE) matrices for the top five AlphaFold2 models of porin A. Low predicted error (blue) indicates high confidence in the relative positioning of residues within domains, whereas higher error (white to red) highlights uncertainty in interdomain orientations.

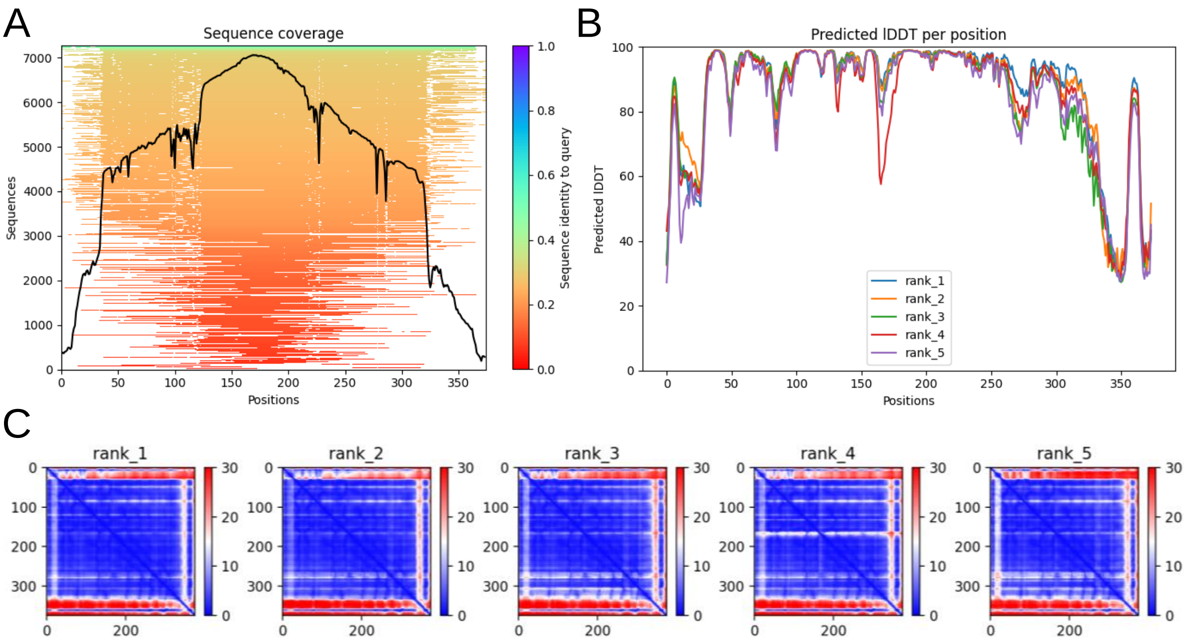

Supplementary figure 6: Quality analysis of the structure prediction for the α/β-hydrolase. (**A**) Multiple sequence alignment of the α/β-hydrolase. Shown are the sequence coverage and sequence identity of homologous structural templates used for the AlphaFold2-based structure prediction. (**B**) pLDDT scores of the α/β-hydrolase. Displayed are the pLDDT scores per amino acid position for the five predicted models. The model with the highest average pLDDT score (blue, rank_1) was selected for further analyses. (**C**) Predicted aligned error (PAE) matrices for the top five AlphaFold2 models of α/β-hydrolase. Low predicted error (blue) indicates high confidence in the relative positioning of residues within domains, whereas higher error (white to red) highlights uncertainty in interdomain orientations.

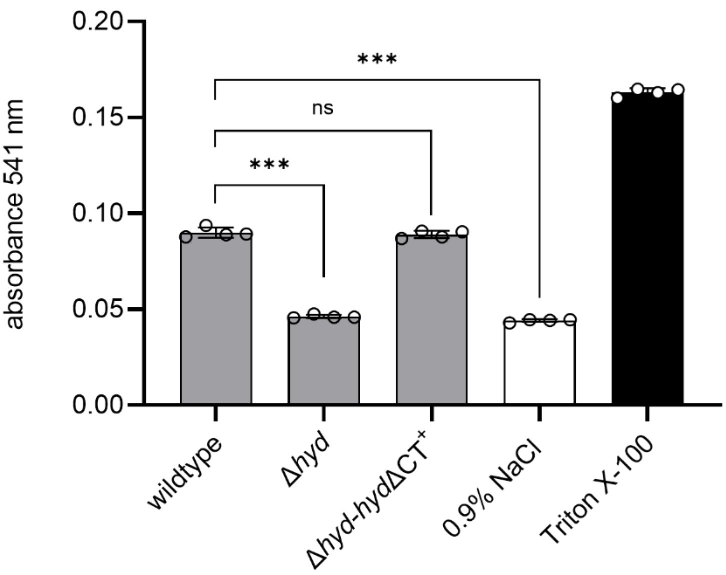

Supplementary figure 7: Hemolytic activity of a truncated α/β-hydrolase variant of *B. bacilliformis.* A truncated version lacking the hydrophobic C-terminal region was cloned under the control of a constitutive promoter into the broad-host-range plasmid pBBR1-MCS and introduced into a *B. bacilliformis* hydrolase deletion mutant. Negative controls (white bar): erythrocytes incubated in 0.9% NaCl without bacteria, positive controls: erythrocytes treated with 1% (v/v) Triton X-100 (black bar). The truncated hydrolase restored hemolytic activity to wild-type levels (grey bars), indicating that removal of the C-terminal region does not impair its function. All samples were run in quadruplicate (n = 4). Data are presented as mean values ± SD. Statistical significance was determined by one-way ANOVA (F (4, 15) = 2,773, $p < 0.001$, $R^2 = 0.999$), followed by Dunnett's post hoc test ($p \geq 0.01$: not significant, ns; $p < 0.001$: significant ***). Two independent experiments have been replicated.

References

1. Stahl, J. et al. *Acinetobacter baumannii* virulence is mediated by the concerted action of three phospholipases D. *PloS one* 10 (9), e0138360 (2015).

Supplementary information: uncropped scans of blots and gels (Figure 4).

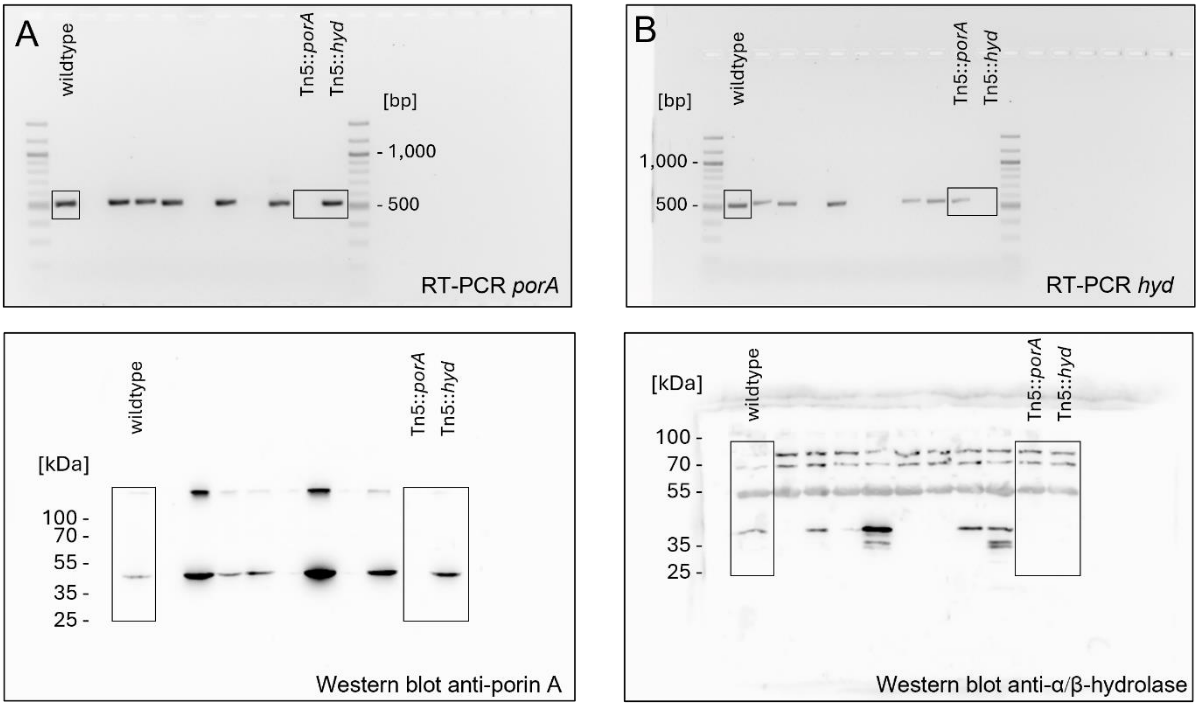

Supplement: Supplementary file 1 — supplementary information [file 41467_2025_66781_MOESM1_ESM.pdf]
